# How do climbing fibers teach?

**DOI:** 10.3389/fncir.2012.00095

**Published:** 2012-11-30

**Authors:** Thomas S. Otis, Paul J. Mathews, Ka Hung Lee, Jaione Maiz

**Affiliations:** Department of Neurobiology and Center for Learning and Memory, Geffen School of Medicine at UCLALos Angeles, CA, USA

**A commentary on**

**A theory of cerebellar cortex**

by Marr, D. (1969). J. Physiol. 202, 437–470.

**A commentary on**

**A theory of cerebellar function**

by Albus, J. S. (1971). Math. Biosci. 10, 25–61.

Four decades ago, Marr and Albus suggested that the climbing fiber (CF) pathway from the inferior olive (IO) to the cerebellum instructs the cellular changes necessary for motor learning (Marr, 1969; Albus, 1971). Subsequent work has confirmed that CFs can drive specific forms of associative motor learning (Gilbert and Thach, 1977; Mauk et al., 1986; Raymond et al., 1996; Jirenhed et al., 2007; Medina and Lisberger, 2008) and has detailed how CFs trigger learning-related forms of synaptic plasticity in Purkinje neurons (PNs) (Linden et al., 1991; Linden and Connor, 1995; Coesmans et al., 2004). Yet, it is widely believed that associative motor learning, such as eyeblink conditioning (Lavond and Steinmetz, 1989; Perrett et al., 1993; Medina and Mauk, 1999; Jorntell and Ekerot, 2002; Ohyama et al., 2006; Shutoh et al., 2006) and vestibulo-ocular reflex (VOR) adaptation (Miles and Lisberger, 1981; Boyden et al., 2004) result from distinct forms of synaptic plasticity that are coordinated at multiple sites within the cerebellar circuit, an idea formalized in several reviews (Raymond et al., 1996; Boyden et al., 2004; Gao et al., 2012). This raises a central question regarding how CFs coordinates plasticity at multiple sites. Simply stated, how do CFs teach?

**Figure 1:**
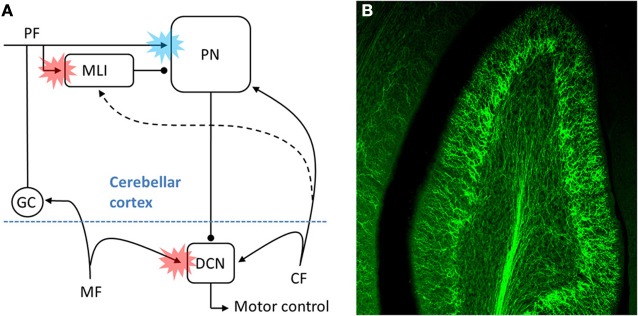
**The CF pathway and associative motor learning. (A)** Schematic of the proposed sites (colored bursts) within the cerebellar circuit where CFs might teach during associative motor learning. The blue burst denotes CF driven LTD of PFs, while the red bursts denote CF-driven LTP of either PF inputs (at MLI) or inputs MF (at DCN). As discussed in the text, for at least two of the three sites the physiological details of the CF input, the underlying cellular mechanisms of plasticity, and the relative contribution to behavioral learning remain to be elucidated. **(B)** ChR2-eYFP expressing CFs are visible in this multi-photon microscopic image of part of folia X in a 300 μm thick rat brain slice.

To address this larger question it is useful to focus on three previously hypothesized sites of associative synaptic plasticity within the cerebellar circuit. At each, the CF is believed to instruct heterosynaptic forms of plasticity by driving changes in the strengths of other excitatory inputs, however, the direction of change triggered by CF activity, i.e., long-term depression (LTD) or long-term potentiation (LTP), is different at each site (Figure 1A). The best described example of CF teaching, and the main focus of the Marr/Albus hypothesis, occurs at the parallel fiber (PF)-to-PN synapse. In mature PNs the single CF input generates a salient and distinctive signal—a cell wide burst termed the complex spike (Eccles et al., 1964)—which instructs heterosynaptic LTD in those PFs that are coactivated with CFs [blue starburst, Figure 1A; (Wang et al., 2000; Hansel et al., 2001; Coesmans et al., 2004; Safo and Regehr, 2008)]. Albus also conjectured that CFs could drive plasticity at a second site, proposing heterosynaptic LTP of PF inputs to a subset of molecular layer interneurons [MLIs; red starburst in cortex, Figure 1A; (Albus, 1971)]. From the perspective of the PN, Albus considered CF enhancement of PF-to-MLI synapses as equivalent to “negative PF synaptic weights.” Considering the fact that PNs spontaneously pacemake at rates up to ~80 Hz (Hausser and Clark, 1997; Raman et al., 1997), PF-to-MLI LTP makes it possible to instruct learned pauses in PN spiking, something that PF LTD on its own cannot accomplish. Although this form of associative plasticity has yet to be demonstrated, there is evocative *in vivo* evidence that indirectly supports it (Jorntell and Ekerot, 2002). The final site at which CFs might instruct plasticity is at mossy fiber (MF)-to-deep cerebellar nucleus neuron (DCN) synapses (red starburst in the deep nuclei, Figure 1A). Much theoretical and experimental work supports the notion that MF-to-DCN synapses strengthen during associative learning (Miles and Lisberger, 1981; Lavond and Steinmetz, 1989; Perrett et al., 1993; Chen et al., 1996; Raymond et al., 1996; Garcia and Mauk, 1998; Medina and Mauk, 1999; Ohyama et al., 2006; Shutoh et al., 2006), and some of these studies indicate that plasticity in the cortex may precede or consolidate plasticity in the DCN (Ohyama et al., 2006; Shutoh et al., 2006; Wulff et al., 2009). The predicted consequence of all three forms of plasticity is to increase DCN excitability in response to particular patterns of MF/PF inputs. While it is generally accepted that CFs drive associative LTD of PFs, it is not clear whether CFs drive the associative forms of LTP during learning (i.e., PF LTP at the MLIs and MF LTP at the DCN). Perhaps relatedly, there is also some debate to whether any one of these forms of plasticity, including PF LTD (Schonewille et al., 2011), are necessary for motor learning.

In an effort to better understand the mechanistic details of how CFs participate in cerebellar learning, we have exploited optogenetic and pharmacological approaches to selectively manipulate CF signals. Using adeno-associated viral delivery of ChR2-eYFP to IO neurons we are able to transfect CFs with high efficiency and specificity in the rat (Figure 1B; Mathews et al., 2012). Optical activation then gives rise to “pure” CF signals generated at the key sites within the cerebellar circuit identified in Figure 1A. This approach shows that MLIs are cooperatively excited by several CFs, giving rise to a robust, CF-driven, feed-forward inhibition that can in turn lead to a transient, synchronous pause in multiple PNs (dashed line, Figure 1A). The CF excitation of MLIs shows cooperativity in part because it can result from the indirect spillover of glutamate from multiple CFs to an MLI, a phenomenon first described by Barbour and colleagues (Szapiro and Barbour, 2007). In this way our observations suggest that MLIs might read out population activity in many CFs (Bell and Kawasaki, 1972; Welsh et al., 1995; Lang et al., 1999; Marshall and Lang, 2009; Mukamel et al., 2009; Ozden et al., 2009), and that synchronous CF input to modules of PNs would result in synchronous pauses in PN spiking. In such a model, CF-dependent pauses in groups of PNs could then serve as proxy teaching signals in the DCN.

In complimentary experiments we have used specific pharmacological tools to manipulate a component of the CF signal, the post-complex spike pause in simple spike firing rate. Two very different compounds (1-EBIO, a positive modulator of calcium activated K^+^ channels, or ZD 7288, an inhibitor of hyperpolarization activated cation channels) were each demonstrated to prolong the post-complex spike pause (Maiz et al., 2012). Either of these drugs infused into the cerebellar cortex during eyeblink conditioning resulted in markedly faster learning (Maiz et al., 2012). We hypothesize that prolongation of the CF-associated pause drives faster learning by facilitating associative LTP of MF inputs to DCN neurons (see Figure 1A). Considering the NMDA receptor dependence of MF to DCN plasticity, it is straightforward to imagine how a pause in descending PN inhibition could associatively drive the types of LTP that have been described *in vitro* (Pugh and Raman, 2006, 2009).

A critical step in understanding cerebellar learning is to explain how CFs, or other teaching signals, coordinate learning-related changes within the circuit. The experiments described here address important questions about the biology of the CF and how it might operate as a teaching signal. Related questions include whether CFs give rise to distinctive postsynaptic signals at those sites in the circuit where they have been hypothesized as teachers, and whether CFs trigger heterosynaptic, associative forms of plasticity that might contribute to learned motor behaviors. The striking anatomical organization of the cerebellar cortex coupled with the remarkable properties of the CF contact on PNs led to the insightful conjecture of Marr and Albus more than 40 years ago. Our observations are consistent with CFs exerting control over multiple sites within the cerebellar circuit, in part through indirect actions read out by MLIs or groups of PNs, a picture that brings to mind the aphorism from the Talmud, *“When you teach your son, you teach your son's son.”* Future experiments utilizing a wide breadth of classical and novel techniques, like those mentioned here will be required to determine just how paternalistic the CF is, and whether it broadens its influence in an analogous way.
